# Protective effects of hydro-alcoholic extract of *Quercus brantii* against lead-induced oxidative stress in the reproductive system of male mice 

**Published:** 2018

**Authors:** Ali Soleimanzadeh, Mehdi Kian, Sajjad Moradi, Farin Malekifard

**Affiliations:** 1 * Department of Theriogenology, Faculty of Veterinary Medicine, Urmia University, Urmia, Iran*; 2 * Department of Pathobiology, Faculty of Veterinary Medicine, Urmia University, Urmia, Iran*

**Keywords:** Antioxidants, Lead, Lipid Peroxidation, Oak Tree, Oxidative Stress, Sperm

## Abstract

**Objective:**

Exposure to heavy metals such as lead (Pb) results in oxidative stress induction in the male reproductive system. Herbal medicine can be utilized as antioxidant agents against oxidative stress. *Quercus brantii* (QB) has shown antioxidant activity in previous studies. The aim of the present study was to evaluate effects of QB hydro-alcoholic extract against Pb-induced oxidative stress in the male mice reproductive system.

**Materials and Methods:**

Forty-two NMRI adult male mice were randomly divided into 7 groups of 6 animals each. Group I was the control group that received no treatment. Group II was the sham group and received 0.2 ml distilled water. Groups III and IV received QB hydro-alcoholic extract 500 and 1000 mg/kg bw, respectively. Group V received Pb 1000 ppm/kg bw. Group VI and VII received Pb 1000 ppm/kg bw and QB extract 500 and 1000 mg/kg bw, respectively. All groups received treatment via oral gavage. After 35 days, sperm parameters (i.e. sperm motility, count and morphology) were evaluated. Levels of sex hormones including LH, FSH, and testosterone, total antioxidant capacity (TAC), superoxide dismutase (SOD) and malondialdehyde (MDA) were measured in animals’ serum.

**Results:**

Exposure to Pb negatively affected sperm parameters (i.e. sperm motility, count and morphology), decreased serum concentrations of sex hormones (i.e. LH, FSH, and testosterone), TAC and SOD activity but increased MDA levels. However, co-administration of 500 and 1000 mg/kg bw QB hydro-alcoholic extract and Pb considerably improved sperm parameters (i.e. sperm motility, count and morphology), increased sex hormones (i.e.LH, FSH, and testosterone), TAC, and SOD activity while decreased MDA levels in animals’ serum.

**Conclusion:**

Administration of QB extracts (Low dose and high dose) is able to protect the male reproductive system of mice against Pb-induced oxidative stress.

## Introduction

Occupational and environmental exposures to heavy metals have been reported to cause harmful effects on human fertility (Ayinde et al., 2012 ▶). One of the most harmful heavy metal present in the environment is lead (Pb) (Patra et al., 2011 ▶). Paint chips/dust, soil, air, water, food, as well as households (e.g. pottery and ceramics), folk remedies, cosmetics, and drugs of abuse are common sources of Pb (Kianoush et al., 2015 ▶). 

One of the major manifestations of Pb toxicity is observed in the male reproductive system (Kakkar & Jaffery, 2005 ▶). Sperm count, motility and activity of spermatozoa could be decreased following Pb exposure (Chowdhury, 2009 ▶). Pb induces oxidative stress by over production of reactive oxygen species (ROS) leading to increases in lipid peroxidation level (El-Nekeety et al., 2009 ▶; Malecka et al., 2009 ▶; Patra et al., 2011 ▶). The negative impacts of oxidative stress on the male reproductive system has been well explained previously (Agarwal et al., 2014 ▶; Aitken & Roman, 2008 ▶).

Since antioxidant compounds can neutralize free radicals and reduce oxidation damages, they can protect organisms from oxidative damages (Packer et al., 2001). Plants are favorable sources of antioxidants because of their natural origin, affordability and fewer side effects (Naik et al., 2003 ▶). 

*Quercus brantii *Lindl. (QB) (locally called “Baloot” in Farsi) from the family Fagaceae, is a small tree widely distributed in Zagros Mountains, Iran. Acorn (locally called “Jaft” in Farsi) is used to treat diarrhea and inflammation in folk medicine (Amin, 1991 ▶; Safary et al., 2009 ▶). Antibacterial (Bajalan et al., 2014 ▶; Tahmouzi, 2014 ▶), antidiabetic (Dogan et al., 2015 ▶), antioxidant (Moradi et al., 2016a ▶; Tahmouzi, 2014 ▶), antiproliferative (Moradi et al., 2016b ▶), and antiviral (Karimi et al., 2017 ▶; Karimi et al., 2013 ▶) effects of QB acorn have been reported in previous studies.

Acorns have high amounts of potassium, iron and zinc (Mohammadzadeh et al., 2013 ▶). They contain considerable amounts of antioxidants like, gallic acid, methyl gallate, p-coumaric acid, ellagic acid, rutin, and quercetin (Çoruh et al., 2014 ▶). Hence, QB acorns are good sources of antioxidants. Heretofore, no study has been reported possible protective and antioxidant role of QB against oxidative stress in the male reproductive system. 

With regard to the antioxidant properties of the QB, the present work was conducted to assess the protective effects of the hydro-alcoholic extract of QB acorns against Pb-induced oxidative stress in the reproductive system of male mice.

## Materials and Methods

**Extract preparation**

Acorns of the QB were collected from the mountains of Sardasht (West Azerbaijan, Iran), and their identity was confirmed in Department of Biology, Faculty of Science, Urmia University (Herbarium No. 4179) by Dr. Zeinali. Then, acorns were washed, air-dried, sliced into small pieces, and grinded to a fine powder. Next, the powder was extracted by percolation using 96% ethanol (Merck, Darmstadt, Germany) and distilled water (50%:50% v:v). After 72 hr, the extract was filtered through Whatman filter No. 40. Then, ethanol was evaporated by a rotator evaporator system and the remaining extract was dried in an oven. Finally, the QB hydro-alcoholic extract was stored at -20°C for *in-vivo* experiments. 

**Animals**

Forty-two NMRI male mice (30±6 g and 8-10 weeks old) were obtained from the animal house of Urmia Medical University, Iran. The animals were housed in plastic (polypropylene) cages and kept under standard laboratory conditions of light/dark cycle (12hr/12hr), temperature (23±2°C) and humidity (70±10%). They were acclimatized for one week before initiation of the study and provided with tap water and standard laboratory diet, *ad libitum*. The study was conducted under the regulations of Animal Ethics Committee in Urmia University (3/TDT/1811, 2016). 

**Experimental design**

Animals were divided into 7 groups of 6 animals each. Group I was the control group and received no treatment. Group II was the sham group and received 0.2 ml distilled water. Groups III and IV received QB extract 500 and 1000 mg/kg bw, (Azizi et al., 2014) respectively. Group V received Pb (lead acetate trihydrate [(C2H3O2)2Pb.3H2O]), Sigma-Aldrich Chemicals Co., St. Louis, USA) 1000 ppm/kg b.w. Group VI and VII received Pb 1000 p.p.m/kg bw and QB extract at the doses of 500 and 1000 mg/kg bw, respectively. 

**Plasma sampling**

After 35 days (Mangoli et al., 2013), all animals were anesthetized then sacrificed by cutting neck vessels. Blood was collected and centrifuged at 3000 g for 15 min. Then, serum was separated and stored at -20° C for biochemical and hormonal evaluations.

**Collection of epididymal sperms**

Sperms were obtained from the cauda epididymis of the testes of each mice. The cauda epididymis was quickly removed and excised into small pieces and placed in a petri dish containing 1 ml of Human Tubal Fluid (HTF) medium (Fernandez et al., 2011) at 37°C (5% CO_2_) for 30 min. 

**Sperm parameters**

Sperm motility, count and morphology were determined through microscopic examination according to WHO laboratory manual for the examination and processing of human semen (World Health Organization, 2010). Sperm smears were obtained from the resulting suspension stained with Eosin-Nigrosin stain and Acridine-Orange for evaluating sperm viability and DNA damages, respectively. An aliquot of sperm suspension was diluted (1:20) with Ham's F10 medium and spermatozoa were transferred into a Neubauer's hemocytometer under a coverslip. Approximately 200 sperms were microscopically examined for each mice. A binocular microscope with 10X eyepieces and 100X oil immersion objective lenses, were used in this study. Abnormally-shaped sperms were recorded randomly and microphotographs were taken if necessary.

**Hormonal assay**

Serum levels of LH and FSH were determined by an enzyme-linked immunosorbent assay (ELISA) using specific commercial kits (Amersham, Buckinghamshire, UK) according to a previous study (Loraine & Bell, 1971).

Serum concentration of testosterone was measured by an enzyme-linked immunosorbent assay (ELISA) as described in the instructions provided by the manufacturer (Demeditec Diagnostics GmbH, Germany). 

**TAC assay**

The TAC of the serum was determined by ferric reducing antioxidant power (FRAP) assay (Benzie & Strain, 1999). Here, 100 µl of serum was added to 1 ml of fresh FRAP reagent (Tripyridyltriazine; Merck, Germany) and incubated at 37°C for 10 min in the dark. The absorbance of the blue-colored complex was read at 595 nm every 20 sec for 10 min. Aqueous solution of Fe II (FeSO_4_.7H_2_O) and appropriate concentrations of freshly prepared ascorbic acid were used as blank and standard solutions, respectively.

**SOD assay**

Serum SOD was determined according to the method reported by Nishikimi et al. (1972) at 560 nm over a 5-min period. The method is based on the ability of the enzyme to inhibit the phenazine methosulphate (Sigma-Aldrich Chemicals Co., St. Louis, USA)-mediated reduction of nitroblue tetrazolium dye.

**Lipid peroxidation**

The rate of lipid peroxidation in serum samples was estimated by determination of malondialdehyde (MDA) levels using thiobarbituric acid reactive substances (TBARS Sigma-Aldrich Chemicals Co., St. Louis, USA) test (Placer et al., 1966).

**Statistical analysis**

Differences between groups were analyzed by one-way analysis of variance (ANOVA), followed by Tukey *post hoc* test by SPSS version 17.0 (IBM Corp., New York, USA). A p value of less than 0.05 was considered significant. Data were expressed as mean±standard error of mean (S.E.M.).

## Results

**Sperm Parameters**

Data in Table 1 shows sperm parameters including sperm count, viability, motility and morphology decrease but TZI, and DNA damage increase in a significant way in Pb group compared to other groups. However, administration of QB significantly improved these reductions in sperm parameters, which the efficacy of concentration of 500 mg/kg.bw better than a concentration of 1000 mg/kg.bw.

**Table 1:** Sperm parameters in different groups.

**Groups**	**Control**	**Pb**	**Control sham**	**QB 500**	**QB 1000**	**Pb + QB 500**	**Pb + QB 1000**
**Sperm count (10** ^6^ **)**	28.25±1.87	12.50±1.54*	27.37±1.43^#^	28.44±0.91 ^#^	27.46±1.38 ^#^	17.44±1.62^*^^†^	15.54±1.39^*^^†^ ●
**Sperm viability (%)**	78.74±1.59	55.19±0.88 *	79.25±1.19 ^#^	80.17±1.30 ^#^	79.60±0.81 ^#^	64.19±1.65^*^^†^	61.29±1.08 ^*^^†^ ●
**Sperm motility (%)**	79.19±1.56	46.19±1.39 *	78.39±1.46 ^#^	80.85±1.57 ^#^	79.91±1.39 ^#^	54.66±1.85^*^^†^	51.56±1.59 ^*^^†^ ●
**Sperm morphology (%)**	87.94±1.60	74.30±0.61^§^	87.77±1.09^†^	87.37±1.40 ^†^	86.69±1.67 ^†^	80.94±1.37^§^^†^	77.34±1.49^§ ^^†^ ●
**Teratozoospermia Index (%)**	1.08±0.46	1.97±0.61 ^§^	1.09±0.46^†^	1.06±0.84 ^†^	1.08±0.48 ^†^	1.75±0.29 ^§^^†^	1.80±0.68 ^§^^†^
**DNA damage (%)**	3.49±0.88	19.55±0.63 *	3.86±0.54 ^#^	3.30±0.57 ^#^	3.35±0.84 ^#^	15.20±1.09^*^^†^	16.39±1.84 ^*^^†^ ●

- Values represent means±SEM (N = 6).

* P<0.001 vs. control;

§ P<0.05 vs. control;

# P<0.001 vs. Pb;

† P<0.05 vs. Pb;

● P<0.05 vs. QB 500. Values represent means±SEM (N = 6).

- QB: *Quercus brantii*; Pb: lead

**Table 2:** Sex hormones levels in different groups.

**Groups**	**Control**	** Pb**	**Control sham**	**QB 500**	**QB 1000**	**PB + QB 500**	**Pb + QB 1000**
**FSH (mIU/ml)**	4.26±0.43	1.69±0.54^*^	4.15±0.32^#^	4.30±0.32 ^#^	4.28±0.25 ^#^	2.39±0.37^†^^#^	1.97±0.64 ^*^^†^^●^
**LH (mIU/ml)**	3.39±0.50	1.52±0.49 ^*^	3.25±0.58 ^#^	3.44±0.38 ^#^	3.42±0.50 ^#^	2.06±0.44 ^†^^#^	1.94±0.72 ^*^^†^^●^
**Testosterone (μmol/L)**	5.86±1.09	3.02±1.87 ^*^	5.65±0.67^#^	6.29±0.57^§^^#^	5.94±1.84 ^#^	4.53±0.82 ^*^^#^	4.08±1.07 ^*^^†^^●^

- Values represent means±SEM (N = 6).

* P<0.001 vs. control;

§ P<0.05 vs. control;

# P<0.001 vs. Pb;

† P<0.05 vs. Pb;

● P<0.05 vs. QB 500. Values represent means±SEM (N = 6).

- QB: *Quercus brantii*; Pb: lead

**Hormonal Assays**

The mean serum concentrations of sex hormones are significantly lower in Pb group compared to all treated (sham and QB groups) and control groups. But, serum concentration of these hormones are significantly higher in groups which simultaneously received Pb and QB compared to the Pb group (Table 2). 

**Antioxidant activity**

As presented in Figures 2 and 3, Pb significantly decreased TAC and SOD levels in Pb group compared to the control group. Co-administration of QB considerably increased TAC and SOD levels compared to Pb groups. No significant differences were observed between other groups (control, control sham and QB 1000).

**Lipid Peroxidation**

A significant increase in MDA level in Pb group was observed compared to other groups (Figure 4). Two groups which were treated with Pb and QB extract at the same time, exhibited significant decreases in MDA levels compared to Pb group. There were no significant differences in MDA level between other groups (control, control sham QB 500 and QB 1000). 

**Figure 1:**
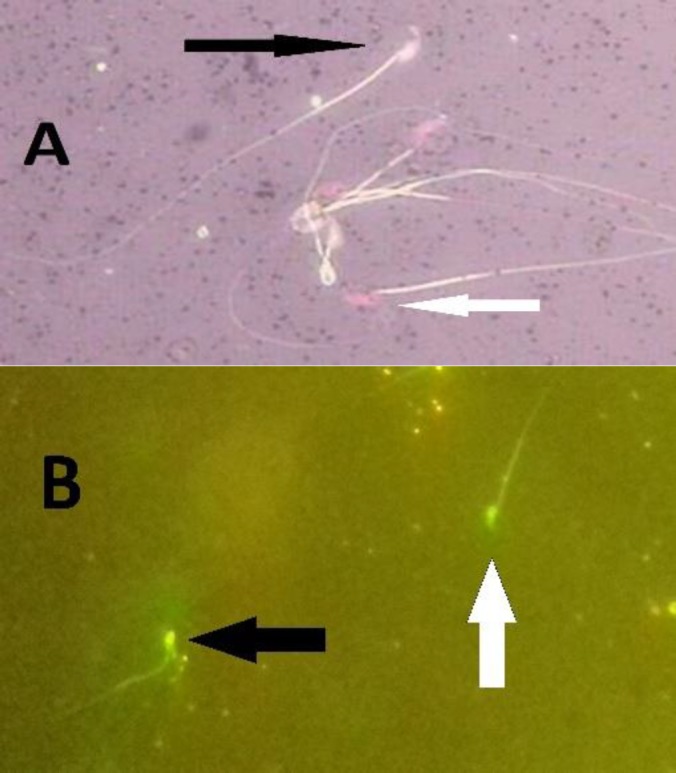
A] Sperm viability group; Black arrow) Viable sperm (colorless), White arrow) Dead sperm (red); (Eosin/nigrosin, 1000X). B] Mice spermatozoa; White arrow) Normal sperm (green); Black arrow) Damaged DNA (yellow); (AO, 400X).

**Figure 2:**
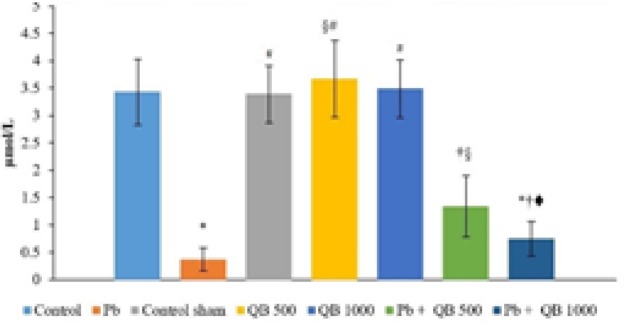
The mean amounts of TAC in different group.

- TAC: Total Antioxidant Capacity; QB: *Quercus brantii*; Pb: lead

- * p<0.001 vs. control; §P<0.05 vs. control; # p<0.001 vs. Pb; †p<0.05 vs. Pb; ●p<0.05 vs. QB 500. Values represent means±SEM (N = 6).

**Figure 3:**
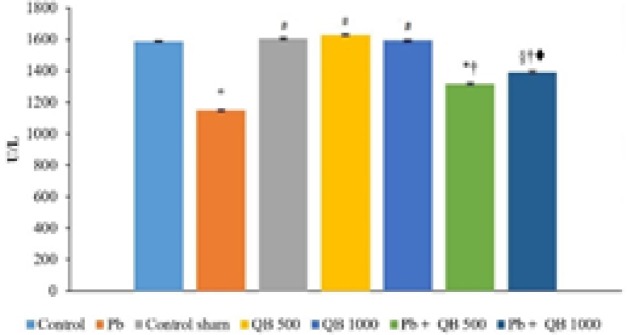
The mean amounts of SOD in different groups.

- SOD: Superoxide dismutase; QB: *Quercus brantii*; Pb: lead.

- * p<0.001 vs. control; §p<0.05 vs. control; # p<0.001 vs. Pb; †p<0.05 vs. Pb; ●p<0.05 vs. QB 500. Values represent means±SEM (N = 6).

**Figure 4:**
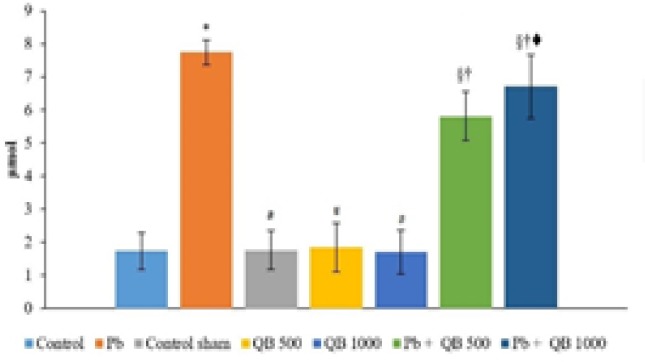
The mean amounts of MDA in different groups.

- MDA: Malonedialdehyde; QB: *Quercus brantii*; Pb: lead

- * p<0.01 vs. control; §P<0.05 vs. control; # p<0.01 vs. Pb; †p<0.05 vs. Pb; ●P<0.05 vs. QB 500. Values represent means±SEM (N = 6).

## Discussion

Herbal medicines are widely used as alternative therapies in the world (Dogan et al., 2015 ▶). In the present study, the protective effects of QB extract against Pb-induced oxidative stress in the reproductive system of male mice were evaluated. Additionally, based on the results, low dose (500 mg/kg.bw) of QB hydro-alcoholic extract is more effective on sperm parameters and sex hormones than high dose (1000 mg/kg.bw) but on the other hand, the high dose has better effects on antioxidant activities and lipid peroxidation than low dose.

Based on our results, Pb reduced sperm quality. Similar to our study, many researchers have reported negative effects of Pb on sperm parameters (Anjum et al., 2017 ▶; Anjum et al., 2011 ▶; Dorostghoal et al., 2014 ▶; Mabrouk & Ben Cheikh, 2014 ▶). The reason of reduction in sperm numbers is due to the ability of Pb in crossing the blood-testis barrier (Creasy, 2001 ▶) which results in impairment of spermatogenesis by induction of oxidative stress and altering normal histology of testes (Elgawish & Abdelrazek, 2014 ▶; EL-Shafai et al., 2011 ▶). Also, Pb binds DNA and damages its double helix structure (Zhang et al., 2014).

In the present study, concentrations of LH and FSH reduced in Pb group which was in line with previous reports (Al-omair et al., 2017 ▶; Ayinde et al., 2012 ▶; Dorostghoal et al., 2014 ▶). Exposure to Pb leads to degenerative changes in endocrine cells of pituitary gland (Hamadouche et al., 2013) which could be the reason of reductions in serum LH and FSH reduction. In contrast with this study, some others have reported that exposure to Pb increases LH and FSH concentrations. This discrepancy between the reports could be due to different concentrations of Pb used, duration of exposure, and the physiological state of the reproductive axis and the testes (Gandhi et al., 2017 ▶). Testosterone concentration of serum declined in Pb group which is similar to previous studies (Al-omair., 2017 ▶; Anjum et al., 2017 ▶; Anjum et al., 2014 ▶; Ayinde et al., 2012 ▶; Dorostghoal et al., 2014 ▶; Mabrouk & Ben Cheikh, 2014 ▶). This occurs as Pb affects the production of testosterone by both lowering expression of steroid enzymes (Thoreux-Manlay et al., 1995 ▶) and decreasing the number of LH receptors in Leydig cells plasma membrane (Kemprinas et al., 1990 ▶).

In this study, TAC decreased considerably in serum of Pb group animals. This reduction in TAC can sensitize the body to oxidative stress which leads to damages in sperm membranes, proteins and DNA. This may explains the reduction of sperm parameters and increases in DNA damage in Pb group. The decrease in SOD in the animals treated with Pb could be due to the interaction between Pb and co-factors of this enzyme including Zn, Cu and Mn and/or by direct blocking of –SH group in SOD (Patra et al., 2011). These results are in accordance with previous reports (Anjum et al., 2017 ▶; Dorostghoal et al., 2014 ▶; Pandya et al., 2010 ▶).

In the present study, levels of MDA increased in Pb group similar to other studies (Anjum et al., 2017 ▶; Dorostghoal et al., 2014 ▶; Pandya et al., 2010 ▶). Lipid peroxidation products are biomarkers of oxidative stress (Niki, 2008 ▶). Owing to high polyunsaturated fatty acid content of sperms, their plasma membranes are highly sensitive to ROS-induced damage and lipid peroxidation (Aitken, 1995 ▶). Lipid peroxidation damages the structure of the lipid matrix in the membrane of sperms and is associated with the loss of motility and defects in membrane integrity (Vernet et al., 2004).

To the best of our knowledge, this is the first study that indicates protective effects of QB on the male reproductive system. The results revealed that QB extract improves sperm parameters which were declined by Pb. Previous research have reported ameliorative effects of herbs on the male reproductive system against Pb-toxicity in animal models (Dorostghoal et al., 2014 ▶; Elgawish et al., 2014 ▶; Sainath et al., 2011 ▶; Sharma et al., 2010 ▶). Our results are in parallel with these reports. The present study indicated that co-administration of QB with Pb partially restore the sex hormones levels to those of the control group. This may be due to the antioxidant effects of QB.

The results of our study demonstrated that QB increased TAC and SOD activity which were also confirmed by Dogan et al. (2015) ▶. The antioxidant effects of QB is due to its phenolic and tannin contents (Moradi et al., 2016a ▶). Strong antioxidants such as gallic acid, p-coumaric acid, ellagic acid, rutin, epicatechin and quercetin are abundantly found in QB (Çoruh et al., 2014 ▶). Administration of QB extract also decreased MDA levels in mice exposed to Pb which is in agreement with data reported by Dogan et al. (2015) ▶ and Mohammadi et al. (2016) ▶.

This study demonstrates that the QB acorn extract protects against Pb_-_induced oxidative stress in the reproductive system by combating oxidative stress. Therefore, QB has the potential to be used as an antioxidant agent.
